# Effect of perioperative steroids application on dysphagia, fusion rate, and visual analogue scale (VAS) following anterior cervical spine surgery: A meta-analysis of 14 randomized controlled trials (RCTs)

**DOI:** 10.3389/fsurg.2022.1040166

**Published:** 2022-11-01

**Authors:** Xiang Zhang, Yi Yang, Yi-Wei Shen, Ke-Rui Zhang, Li-Tai Ma, Hao Liu

**Affiliations:** Department of Orthopedics, Orthopedic Research Institute, West China Hospital, Sichuan University, Chengdu, China

**Keywords:** steroid, dysphagia, anterior cervical spine surgery, rct, meta-analysis steroid, meta-analysis introduction

## Abstract

**Objective:**

To conduct a high-level meta-analysis of the RCTs to evaluate perioperative steroids use in the management of fusion rate, dysphagia, and VAS following anterior cervical spine surgery for up to 1 year.

**Methods:**

We searched the database PubMed, EMBASE, Web of Science, Cochrane Library, Google Scholar, Ovid, and ClinicalTrials.gov without time restriction to identify RCTs that evaluate the effectiveness of perioperative steroids after anterior cervical spine surgery. A subgroup analysis was undertaken to investigate the effects of intravenous and local steroids. This study was registered in the PROSPERO database prior to initiation (CRD42022313444).

**Results:**

A total of 14 RCTs were eligible for final inclusion. This meta-analysis showed that steroids could achieve lower dysphagia rate (*p* < 0.001), severe dysphagia rate within 1 year (*p* < 0.001), lower VAS scores at both 1 day (*p* = 0.005), 2 weeks (*p* < 0.001) and shorter hospital stay (*p* = 0.014). However, there was no significant difference between the two groups regarding operation time (*p* = 0.670), fusion rates (*p* = 0.678), VAS scores at 6 months (*p* = 0.104) and 1 year (*p* = 0.062). There was no significant difference between intravenous and local steroid administration regarding dysphagia rates (*p* = 0.82), fusion rate (*p* = 1.00), and operative time (*p* = 0.10).

**Conclusion:**

Steroids intravenously or locally following anterior cervical spine surgery can reduce incidence and severity of dysphagia within 1 year, VAS score within 2 weeks, and shorten the length of hospital stay without affecting fusion rates, increasing the operating time, VAS score at 6 months and 1 year.

## Introduction

Since first introduced in 1958 by Cloward (1), Robinson and Smith (2), anterior approach has become the standard approach in the treatment of spondylotic radiculopathy and myelopathy with demonstrated long-term clinical success. However, it is associated with complications such as dysphagia, presumably due to local tissue swelling, intraoperative excessive retraction, and laryngeal nerve palsy. Rates of postoperative dysphagia ranged in frequency from 1.7% to 67% according to previous reports (3–6). Dysphagia after ACDF has raised concerns about increasing morbidity, duration of hospitalization, and medical costs (7).

Many measures have been investigated to decrease the incidence of dysphagia and decreased cuff pressure and plate prominence are just a few (8–11). One promising therapeutic intervention is the use of perioperative steroids (12–14). In some studies, the steroid has resulted in decreased incidence and severity of dysphagia (13, 15). However, the effect of steroids has been equivocal in other studies (16). In addition to inconsistent results for dysphagia, there is concern about the adverse effects of steroids, such as delayed time to fusion (12). From the surgeon's point of view, solid bony fusion is of critical importance in the achievement of expected outcomes following anterior cervical spine surgery. Delayed bony fusion or even non-union after surgery greatly increases the risk of revision (17). In addition, it has been reported that steroids can reduce postoperative pain by reducing the inflammatory response (18). Nevertheless, the duration of this effect still remains controversial.

Considering these issues, it is important to perform a systematic review and meta-analysis to provide clear advice concerning the accurate effect of steroids on the incidence and severity of dysphagia, fusion rate and VAS score. Moreover, a subgroup analysis was needed to compare the effects of intravenous and local steroids as a consensus on the use of intravenous and local injections has not yet been reached.

## Methods

This systematic review was conducted following the Preferred Reported Items for Systematic reviews and Meta-Analysis (PRISMA) guidelines, the Cochrane Collaboration recommendations and AMSTAR (Assessing the methodological quality of systematic reviews) (19, 20), and the study protocol was registered in the international open-access Prospective Register of Systematic Reviews (PROSPERO, number: CRD42022313444) prior to data retrieval.

### Search strategy

A comprehensive literature search was conducted on PubMed, EMBASE, Web of Science, Cochrane Library, Google Scholar, Ovid, and ClinicalTrials.gov from inception to February 19, 2022. Search terms included both entry terms and medical descriptors/MeSH terms such as “Glucocorticoids”, “Steroids”, “Methylprednisolone”, “Dexamethasone”, “anterior cervical discectomy and fusion”, “Anterior cervical surgery”, “Anterior cervical fusion”, “Anterior Cervical Corpectomy and Fusion”. Supplementary File S1 summarizes the search strategy used in each database.

### Assessment of eligibility

Studies satisfying the following criteria were included: (1) population: adults with spondylotic radiculopathy and myelopathy undergoing anterior cervical spine surgery; (2) intervention: perioperative intravenous or local steroids administration; (3) comparison: placebo vs. steroids; (4) main outcomes: the event number of dysphagia, visual analog scale (VAS) at postoperative 1 day, 2 weeks, 6 months and 1 year, fusion rates at 1 year; (5) study design: RCT design.

The following studies were excluded: (1) Letters, editorials, conference abstracts, systematic reviews or meta-analyses, consensus statements, guidelines; (2) Had insufficient data this meta-analysis required; (3) Contained comparisons with other comparison protocols; (4) Full-text was not available.

### Data extraction

Data extraction was conducted by two independent reviewers using a piloted and standardized data extraction form. Any disagreements were resolved by mutual consensus. The following data from each included study were retrieved: (1) Study characteristics: authors' information, publication year; (2) Patients' characteristics: size of each group, mean age, male-to-female ratio; (3) Intervention: route of administration and dose; (4) Outcomes: dysphagia events, fusion rate, VAS score, operation time, length of hospital stay.

### Risk of bias and quality assessment

The quality and risk of bias were assessed by two independent reviewers using the Cochrane Handbook for Systematic Reviews of Interventions (20). Any disagreements were resolved by mutual consensus. This quality evaluation system includes seven domains: random sequence generation, allocation concealment, blinding of participants and personnel, blinding of outcome assessment, incomplete outcome data, selective outcome reporting, and other bias. Each domain was assessed as low, unclear, or high risk. Risk of bias graphs were plotted using the Revman software (version 5.3). The results of outcomes were assessed the quality of evidence by the Grading of Recommendations Assessment, Development and Evaluation (GRADE) under the software GRADE profiler (https://gradeprofler.sofware.informer.com/download/).

### Statistical analysis

We used Stata 14.0 for statistical analysis. Mean difference with 95% confidence intervals (CIs) was used to evaluate continuous data, and odds ratio was used for dichotomous data. *p* value was calculated and documented for each outcome measure. Statistical significance was defined as a *p* value less than 0.05 (*p* < 0.05).

Statistical heterogeneity was assessed using the *I*^2^ test. The *I*^2^ statistic describes the percentage of variation in each study due to heterogeneity rather than chance, while *I*^2^ values of 0%–25%, 25%–50%, 50%–75%, and >75% represent very low, low, medium, and high heterogeneity, respectively (21). A random-effect model was applied when the *I*^2^ value was over 50%, and a fixed-effect model was applied conversely.

In addition, a subgroup analyses by the route of administration (Local vs. Intravenous) was performed to further evaluate the effects of intravenous and local steroids. A sensitivity analysis that excluding studies one by one was performed to investigate the effect of steroid intervention on evaluation indicators.

## Results

### Search results

**Figure 1:**
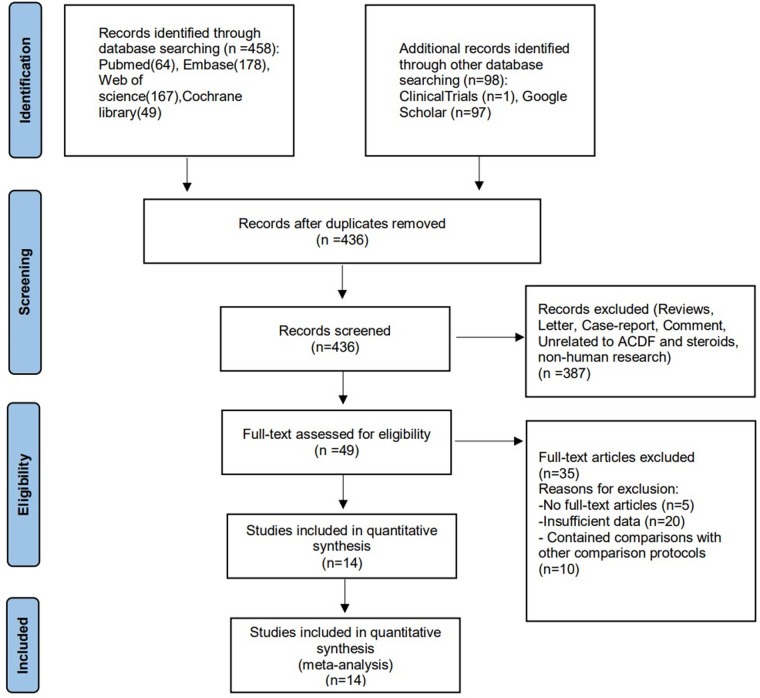
Flow diagram of the included studies.

The systematic literature search initially identified 436 potentially eligible articles from PubMed, Embase, Web of Science, Cochrane Library, Google Scholar, and ClinicalTrials.gov (Figure 1). After excluding 120 duplicates, screening of the remaining 436 titles and abstracts yielded 49 potentially eligible articles. After full-text reviews of the 49 provisionally eligible articles, 35 articles were excluded due to no access to full-text (5), contained insufficient data (20), contained comparisons with other comparison protocols (10). Finally, 14 articles were included in this present systematic review and meta-analysis.

### Characteristics of the included studies

Details of study demographics of steroid-administered patients, details of the administration of the steroids, and steroids effects assessment after anterior cervical fusion are summarized in Table 1, 2. All the 14 articles (16, 18, 22–33) were prospective randomized controlled trials that were graded as the level of evidence 1, and three of them were double-blinded studies (22, 24, 30). A total of 1,181 patients were enrolled across all 14 randomized controlled studies. In total, 252 patients received intravenous steroids, 310 patients received topical steroids, and 619 patients served as controls. The corticosteroid treatment arms utilized IV dexamethasone (16, 22, 28, 29) or methylprednisolone (31, 33) or local injection of methylprednisolone (24–27, 30, 32) or triamcinolone (18, 23, 28).

**Table 1:** The characteristics of the included studies.

Study (year)	Country	Surgery type	Experimental data			Control data			Outcomes recorded	Follow-up
			Patients	Mean age	Male: Female	Patients	Mean age	Male: Female		
Cui et al., 2019	USA	49 ACDF, 8 ACCF, 1 hybrid, and 6 single-level CDA	33	53.2	13:20	31	50.3	15:16	Bazaz dysphagia score, Dysphagia Symptom Questionnaire, fusion rate	12 months
Dahapute et al., 2020	India	1 and 2-level ACDF	25	50.4	19:6	25	50.4	19:6	PSTS, VAS, mJOA, NDI, fusion rate	12 months
Edwards et al., 2016	USA	1, 2, and 3-level ACDF	27	54	11:16	23	54.5	9:14	Bazaz scale, average dysphagia scores, operation time, length of hospital stay	28 days
Grasso et al., 2019	Italy	1 and 2-level ACDF	35	46.1	18:17	35	45.5	17:18	Bazaz scale, VAS, operation time	12 months
Hasani Barzi et al., 2016	Iran	1, 2, and 3-level ACDF	20	50.3	8:12	20	48.3	8:12	PSTS, S/V ratio, VAS	10 days
Haws et al., 2018	USA	1, 2, and 3-level ACDF	55	49.4	31:24	49	50.6	30:19	Mean SWAL-QOL score, mean swelling index, mean air index, VAS, operation time, length of hospital stay	12 weeks
Jenkins et al., 2018	USA	1, 2, and 4-level ACDF	29	55.6	15:14	21	11:10	14:24	Bazaz scale, EAT-10, VHI-10, VAS, fusion rate	12 months
			25	14:24	14:11	21	11:10	14:24		
Jeyamohan et al., 2015	USA	2, 3, 4 and 5-level ACDF	56	54	33:23	56	55	27:28	Bazaz scale, mJOA, FOSS score, ODI score, SF-12 PCS score, SF-12 MCS score, fusion rate, VAS	24 months
Kim et al., 2021	USA	2, 3, 4-level ACDF	56	58.1	27:29	53	58.4	29:24	Eat-10, SWAL-QOL, NDI, operative time, length of hospital stay	1 month
Lee et al., 2011	Korea	1 and 2-level ACDF	25	54.3	18:9	25	50.9	14:7	PSTS, fusion rate, VAS, NDI	22 months
Nam et al., 2013	Korea	1-level ACDF	20	45.6	14:6	22	48.8	16:6	PSTS, VAS, operation time	5 days
			20	46.9	11:9	22	48.8	16:6		
Seddighi et al., 2017	Iran	1, 2, and 3-level ACDF	38	49.3	18:20	38	50.2	16:22	Bazaz scale, PSTS, S/V ratio, VAS, operative time, length of hospital stay	6 months
Song et al., 2014	Korea	≥3-level ACDF	20	59.9	14:06	20	57.3	16:04	Bazaz scale, PSTS, operative time, length of hospital stay	5 days
Pedram et al., 2003	France	1, 2, and 3-level ACDF and ACCF	78	47	Not reported	158	47	Not reported	Throat lesions, operative time	36 h

ACDF, anterior cervical discectomy and fusion; ACCF, anterior cervical corpectomy decompression and fusion; CDA, cervical disc arthroplasty; PSTS, prevertebral soft-tissue swelling; SWAL-QOL, quality of life in swallowing disorders; VAS, visual analog scale; NDI, neck disability index; mJOA, modified Japanese Orthopedic Association Score; S/V, The ratio of prevertebral soft tissue thickness to mid anteroposterior vertebral body; EAT-10, Eating Assessment Tool-10; VHI-10, Voice Handicap Index-10.

**Table 2:** The intervention administration methods, steroid dose and frequency in each included study.

Study	Intervention administration method		Dose		Frequency	
	Steroid group	Control group	Steroid group	Control group	Steroid group	Control group
Cui et al., 2019	Intravenous application	Intravenous application	0.3 mg/kg dexamethasone preoperatively, 0.15 mg/kg dexamethasone postoperatively	Equivalent of saline	1 dose of 0.3 mg/kg preoperatively, 0.15 mg/kg every 8 h for 2 doses postoperatively	2 dose of 0.3 mg/kg preoperatively, 0.15 mg/kg every 8 h for 2 doses postoperatively
Dahapute et al., 2020	Local application	Local application	40 mg triamcinolone	Equivalent of saline	Once intraoperatively	Once intraoperatively
Edwards et al., 2016	Local application	Local application	40 mg Depo-medrol	Equivalent of saline	Once intraoperatively	Once intraoperatively
Grasso, 2019	Local application	Local application	40 mg methylprednisolone	200 ml saline	Once intraoperatively	Once intraoperatively
Hasani Barzi et al., 2016	Local application	None	80 mg methylprednisolone	None	Once intraoperatively	None
Haws, 2018	Local application	Local application	40 mg Depo-medrol	Equivalent of saline	Once intraoperatively	Once intraoperatively
Jenkins et al., 2018	Local application	None	40 mg triamcinolone	None	Once intraoperatively	None
	Intravenous application	None	10 mg dexamethasone	None	Once intraoperatively	None
Jeyamohan et al., 2015	Intravenous application	Intravenous application	0.2 mg/kg dexamethasone intraoperatively, 0.06 mg/kg dexamethasone postoperatively	Equivalent of saline	1 dose of 0.2 mg/kg intraoperatively, 0.06 mg/kg every 6 h for the first 24 h	1 dose of 0.2 mg/kg intraoperatively, 0.06 mg/kg every 6 h for the first 24 h
Kim, 2021	Local application	None	40 mg methylprednisolone	None	Once intraoperatively	None
Lee et al., 2011	Local application	None	40 mg triamcinolone	None	Once intraoperatively	None
Nam et al., 2013	Intravenous application	Intravenous application	10 mg dexamethasone intraoperatively, 5 mg dexamethasone postoperatively	Equivalent of saline	1 dose of 10 mg intraoperatively, 5 mg on postoperative day 1 and day 2, respectively	1 dose of 10 mg intraoperatively, 5 mg on postoperative day 1 and day 2, respectively
	Intravenous application	Intravenous application	20 mg dexamethasone intraoperatively, 10 mg dexamethasone postoperatively	Equivalent of saline	1 dose of 20 mg intraoperatively, 10 mg on postoperative day 1 and day 2, respectively	1 dose of 20 mg intraoperatively, 10 mg on postoperative day 1 and day 2, respectively
Seddighi, Afsoun et al., 2017	Local application	Local application	80 mg methylprednisolone	200 ml saline	Once intraoperatively	Once intraoperatively
Song et al., 2014	Intravenous application	None	250 mg methylprednisolone	None	250 mg and every 6 h for the first 24h	None
Pedram et al., 2003	Intravenous application	None	1 mg/kg methylprednisolone	None	1 mg/kg and every 12 h for the first 24h	None

### Quality assessment to risk of bias

**Figure 2:**
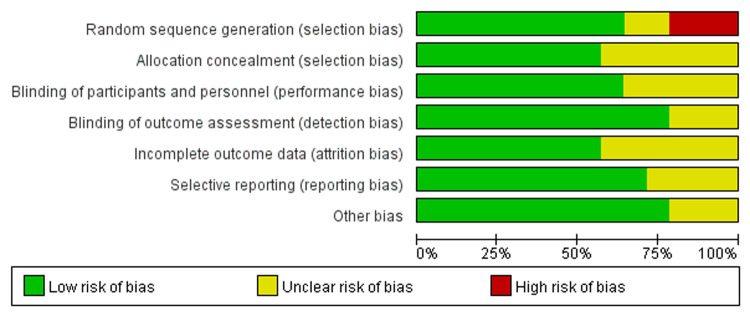
Risk of bias graph.

**Figure 3:**
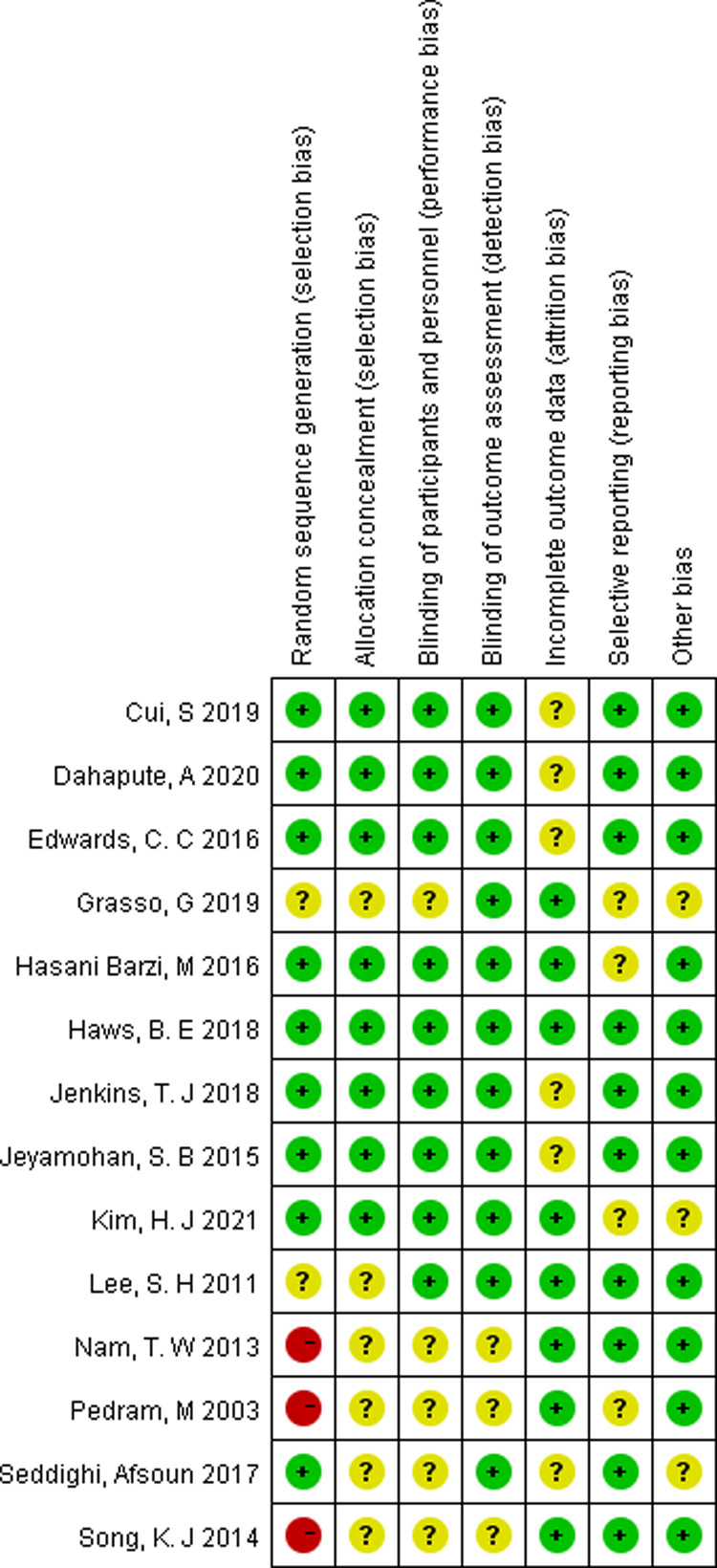
Risk of bias summary of randomized controlled trials.

Two independent reviewers evaluated the quality of 14 RCTs according to the criteria of the Cochrane Collaboration for Systematic Reviews and any disagreements were solved through discussion and consensus. Three studies were found to have a “high” risk of bias, primarily attributed to the randomization process. The overall risk of bias of the included studies was determined to be low (Figures 2, 3).

### Quality of evidence assessment by GRADE

The results of dysphagia event, Bazaz stratification of severity of dysphagia, fusion rate, VAS, operation time and length of hospital stay were assessed the quality of evidence by GRADE. The results qualities of VAS were low, and dysphagia event, Bazaz stratification of severity of dysphagia, fusion rate, operation time and length of hospital stay were moderate. None of high quality evidence was found in above outcomes (Table 3).

**Table 3:** GRADE assessment of the level of evidence for all included studies.

Quality assessment							No of patients		Effect	Quality	Importance
No of studies	Design	Risk of bias	Inconsistency	Indirectness	Imprecision	Other considerations	Steroids	Control	Relative (95% CI) absolute		
**Dysphagia events (follow-up 1 year; assessed with, Dysphagia events)**											
8	Randomized trials	Serious^a^	No serious inconsistency	No serious indirectness	No serious imprecision	None	231/723 (32%)	375/729 (51.4%)	OR 0.34 (0.26 to 0.46)	⊕⊕⊕O MODERATE	CRITICAL
**Dysphagia events (moderate + severe) (follow-up 1 year; assessed with, Bazaz stratification)**											
5	Randomized trials	Serious^b^	No serious inconsistency	No serious indirectness	No serious imprecision	None	77/535 (14.4%)	138/469 (29.4%)	OR 0.21 (0.13 to 0.34)	⊕⊕⊕O MODERATE	CRITICAL
**Fusion rate (follow-up 1 year; assessed with, Fusion events)**											
6	Randomized trials	No serious risk of bias	No serious inconsistency	Serious^c^	No serious imprecision	None	166/191 (86.9%)	154/175 (88%)	OR 0.87 (0.46 to 1.65)	⊕⊕⊕O MODERATE	CRITICAL
**VAS score (follow-up 1 year; measured with, VAS)**											
7	Randomized trials	Serious^d^	Serious^e^	No serious indirectness	No serious imprecision	None	532	484	WMD −1.52 (−2.01 to −1.04)	⊕⊕OO LOW	CRITICAL
**Operation time (measured with, time)**											
7	Randomized trials	No serious risk of bias	Serious^f^	No serious indirectness	No serious imprecision	None	329	400	WMD −2.15 (−5.22 to 0.92)	⊕⊕⊕O MODERATE	IMPORTANT
**Length of hospital stay (measured with, time)**											
4	Randomized trials	No serious risk of bias	Serious^g^	No serious indirectness	No serious imprecision	None	169	160	SMD −0.42 (−0.76 to −0.09)	⊕⊕⊕O MODERATE	IMPORTANT

^a^ Pedram, 2003 and Song may have selection bias.

^b^ Song, 2014 may have selection bias.

^c^ The standard of fusion varied.

^d^ Nam, 2013 may have selection bias.

^e^ I-squared = 93.8%.

^f^ I-squared = 78.6%.

^g^ I-squared = 54.4%.

### Results of meta-analysis

#### The use of steroids for dysphagia event from postoperative 1 day to 1 year

**Figure 4:**
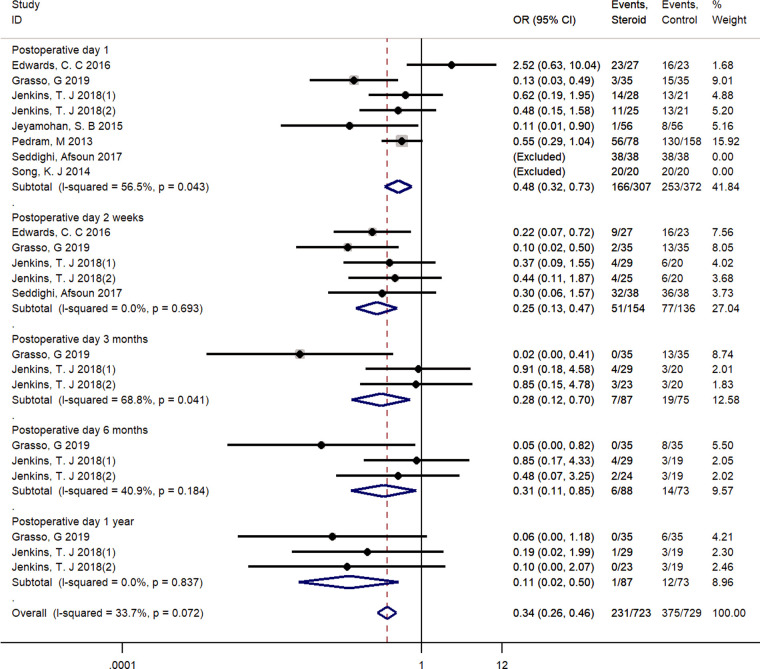
Forest plot of dysphagia events.

The most commonly used assessment tool for dysphagia was the Bazaz scale (25, 28, 29, 31–33). One study used its modified version, the Modified Dysphagia Scoring System (MDSS) (24). The pooled outcomes showed that steroid use achieved significantly lower dysphagia rates compared with the incidence in the control group (1 day, OR = 0.48, 95% CI: 0.32–0.73, 2 weeks, OR = 0.25, 95% CI: 0.13–0.47; 3 months, OR = 0.28, 95% CI: 0.12–0.70; 6 months, OR = 0.31, 95% CI: 0.11–0.85; 1 year, OR = 0.11, 95% CI: 0.02–0.50). With a fixed-effect model, a low heterogeneity among these studies was found in the pooled outcomes (*I*^2^ = 33.7%, *p* = 0.072) (Figure 4).

#### Bazaz stratification of severity of dysphagia (moderate + severe) from postoperative 1 day to 1 year

**Figure 5:**
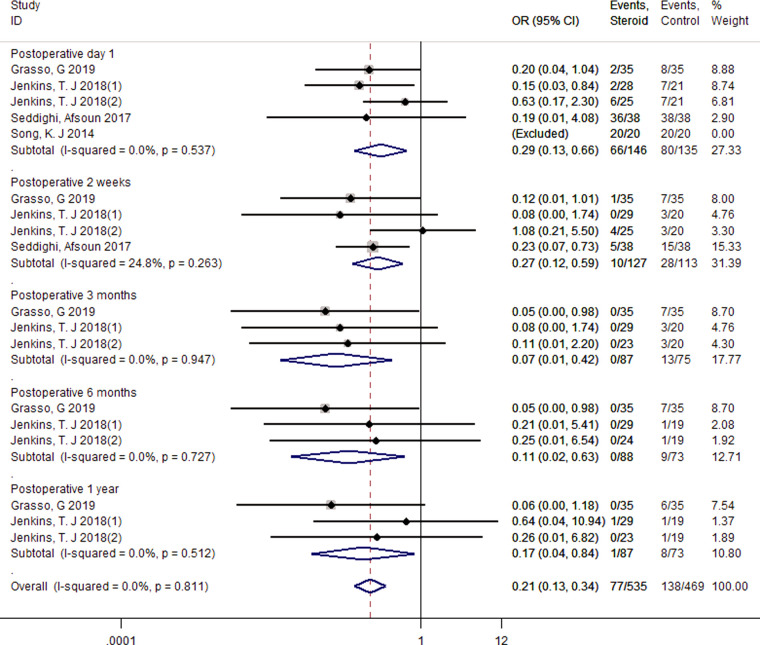
Forest plot of dysphagia events (moderate + severe) according to Bazaz stratification.

A fixed-effect model was used to pool the total moderate and severe Bazaz stratification because there was no significant heterogeneity across four studies (*I*^2^ = 0.00%, *p* = 0.811) (25, 28, 32, 33). The pooled analysis revealed less moderate and severe events in the steroid group compared with the control group within 1 year after surgery (1 day, OR = 0.29, 95% CI: 0.13–0.66; 2 weeks, OR = 0.27, 95% CI: 0.12–0.59; 3 months, OR = 0.07, 95% CI: 0.01–0.42; 6 months, OR = 0.11, 95% CI: 0.02–0.63; 1 year, OR = 0.17, 95% CI: 0.04–0.84) (Figure 5).

#### The use of steroids for fusion rate at 1-year follow-up

**Figure 6:**
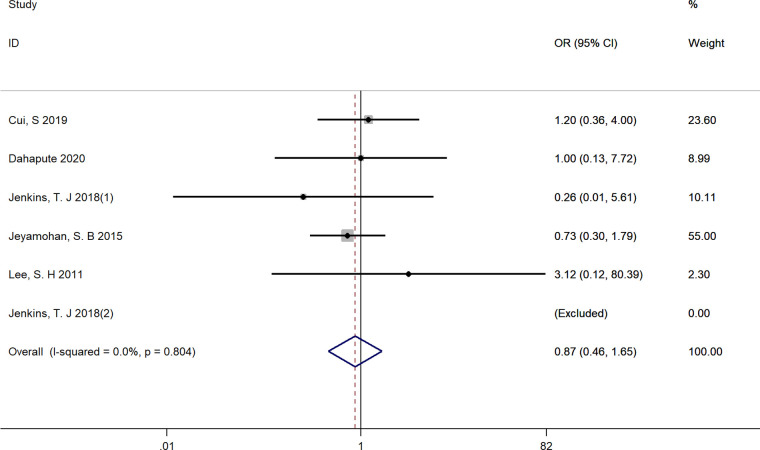
Forest plot of fusion rate at 1-year follow-up time.

Five studies reported numbers of fusion events at 1-year follow-up time and were included (18, 22, 23, 28, 29). There existed no significant difference between groups regarding fusion rate (OR = 0.87, 95% CI: 0.46–1.65), and no significant heterogeneity among these studies was found with a fixed-effect model (*I*^2^ = 0.0%, *p* = 0.999) (Figure 6).

#### The use of steroids for VAS from postoperative 1 day to 1 year

**Figure 7:**
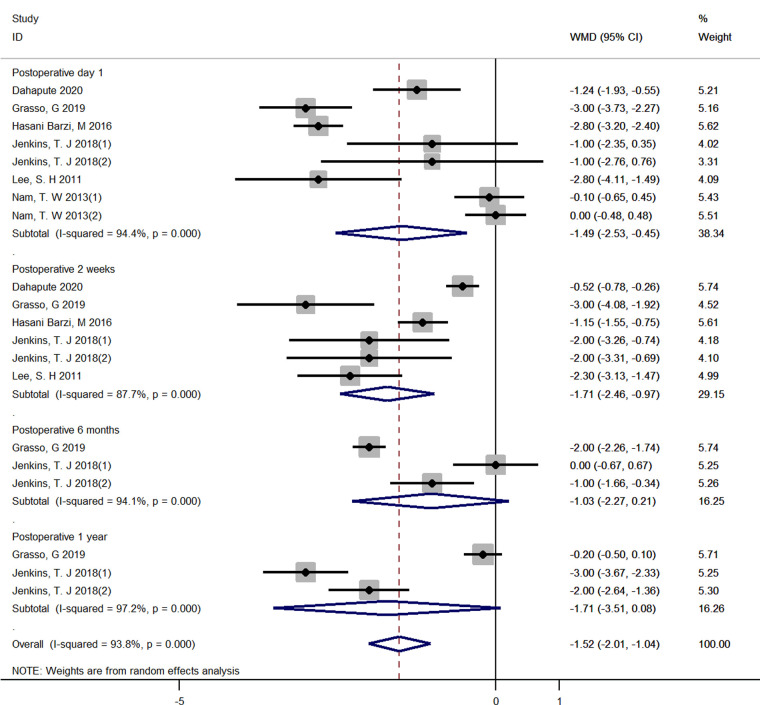
Forest plot of VAS score.

Six RCTs reported the detailed VAS score and were included (16, 18, 23, 25, 26, 28). A random-effect model was applied due to the high heterogeneity (*I*^2^ = 93.4%, *p* < 0.001). A significant decrease regarding VAS score in the steroid group was observed compared with that in the control group at both 1 day, 2 weeks after surgery (1 day, WMD = −1.49, 95% CI: −2.53 to −0.45; 2 weeks, WMD = −1.71, 95% CI: −2.46 to −0.97). However, Pooled analysis revealed no significant difference in the VAS score between two groups at both 6 months and 1 year after surgery (6 months, WMD = −1.03, 95% CI: −2.27 to 0.21; 1 year, WMD = −1.71, 95% CI: −3.51 to 0.08) (Figure 7).

#### Operation time

**Figure 8:**
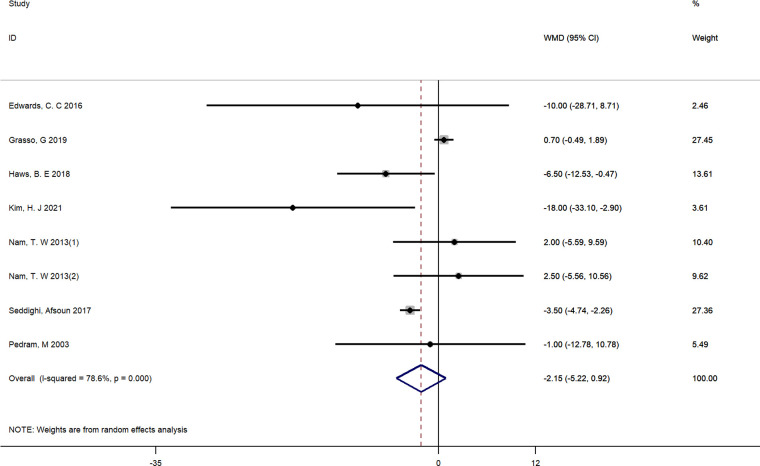
Forest plot of operation time.

Seven studies reported the detailed operation time and were included (16, 24, 25, 27, 30–32). There was significant heterogeneity between studies (*I*^2^ = 78.6%, *p* < 0.01), and a random-effect model was adopted. Pooled results demonstrated that there was no significant difference between groups in operating time (WMD = −2.15, 95% CI: −5.22 to 0.92) (Figure 8).

#### Length of hospital stay

**Figure 9:**
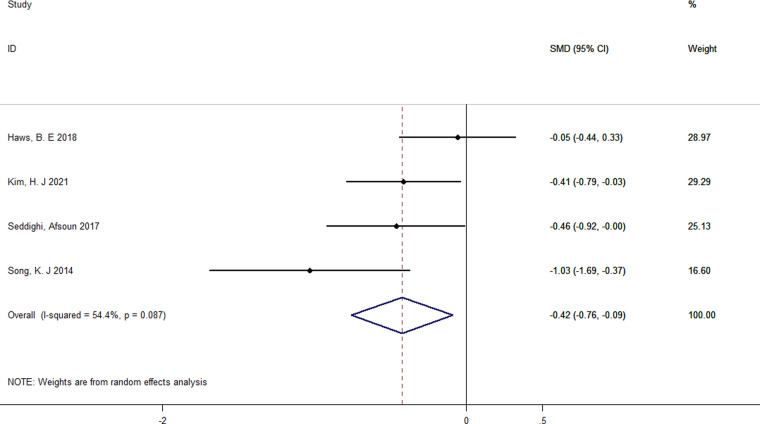
Forest plot of length of hospital stay.

Four studies reported the detailed length of hospital stay and were included (27, 30, 32, 33). A random-effect model was used because the heterogeneity across the three studies was high (*I*^2^ = 54.4%, *p* = 0.087). Pooled results demonstrated a significant reduction in the length of hospital stay compared with that in the control group (SMD = −0.42; 95% CI: −0.76 to −0.09) (Figure 9).

#### Subgroup analysis

**Figure 10:**
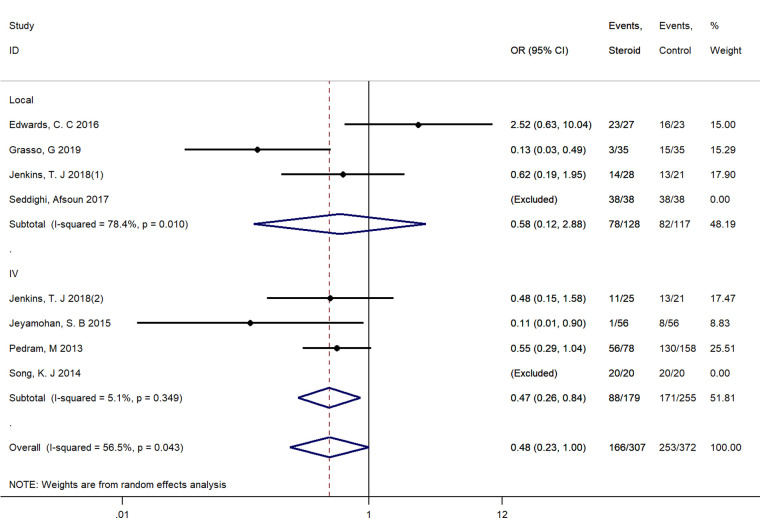
A subgroup analysis between intravenous and local steroid administration regarding dysphagia rates. IV, intravenous.

**Figure 11:**
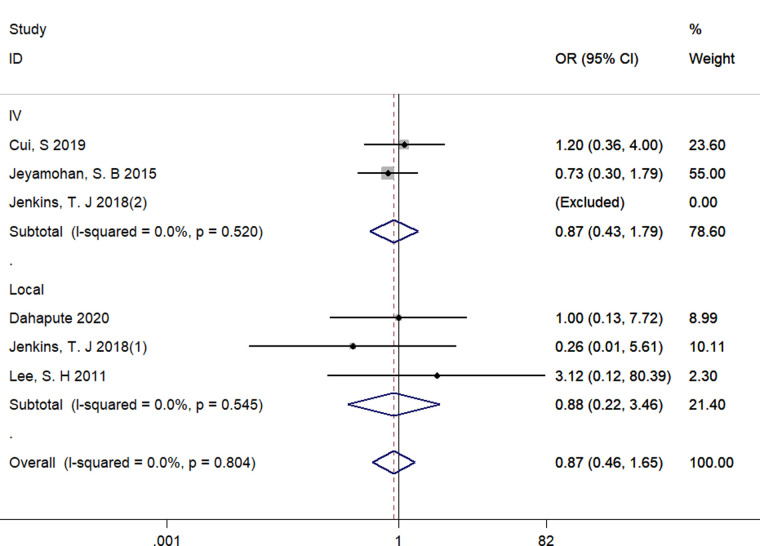
A subgroup analysis between intravenous and local steroid administration regarding fusion rates. IV, intravenous.

**Figure 12:**
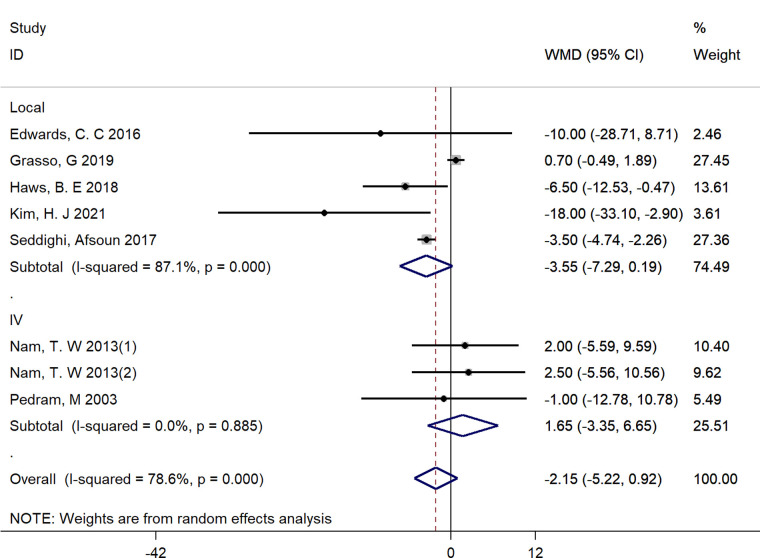
A subgroup analysis between intravenous and local steroid administration regarding operation time. IV, intravenous.

**Figure 13:**
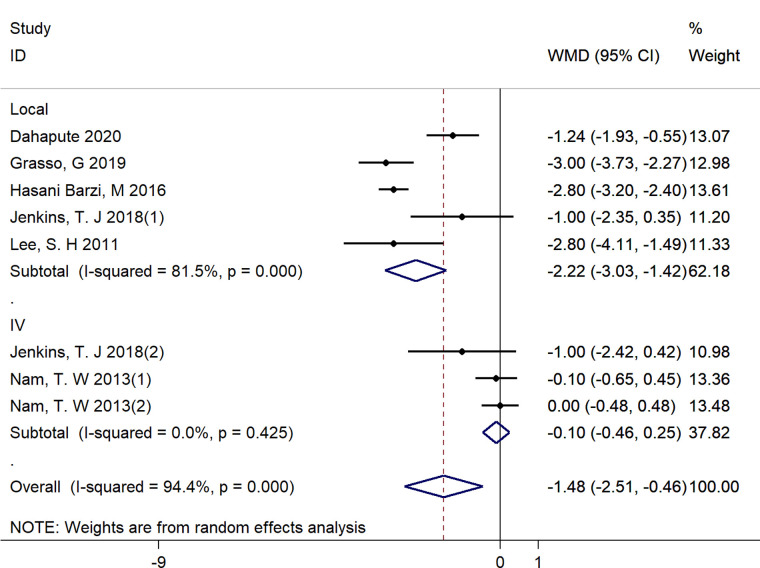
A subgroup analysis between intravenous and local steroid administration regarding VAS score at postoperative 1 day. IV, intravenous.

We performed subgroup analyses by the route of administration (Local vs. Intravenous). Due to the limited number of included studies, we only have sufficient data exploring the effect of local and intravenous application of steroids on dysphagia rates at postoperative 1 day, VAS score at postoperative 1 day, fusion rate and operative time. There was no significant difference between intravenous and local steroid administration regarding dysphagia rates (Local: OR = 0.58, 95% CI: 0.12 to 2.88 vs. Intravenous: OR = 0.47, 95% CI: 0.26 to 0.84, *p* = 0.82, Figure 10), fusion rate (Local: OR = 0.88, 95% CI: 0.22 to 3.46 vs. Intravenous: OR = 0.87, 95% CI: 0.43 to 1.79, *p* = 1.00, Figure 11), and operation time (Local: WMD = −3.55, 95% CI: −7.29 to 0.19 vs. Intravenous: WMD = 1.65, 95% CI: −3.35 to 6.65, *p* = 0.10, Figure 12). However, there existed a significant difference between intravenous and local steroid administration regarding VAS score at postoperative 1 day (Local: WMD = −2.22, 95% CI: −3.03 to −1.42 vs. Intravenous: WMD = −0.10, 95% CI: −0.46 to 0.25, *p* < 0.001, Figure 13).

#### Sensitivity analyses and publication bias

Through the sensitivity analyses, we found that excluding studies one by one did not significantly alter the effect of steroid intervention on evaluation indicators. We did not perform the funnel plot to illustrate the publication bias of the primary outcome because less than 10 articles were included in quantitative analysis of a single outcome.

## Discussion

Anterior cervical surgery has been wildly accepted as the gold standard surgical treatment for patients with cervical disc disease who failed conservative measures (34, 35). Despite the satisfactory clinical outcomes of anterior cervical surgery, up to 79% of patients experienced postoperative dysphagia. Our meta-analysis of 14 RCTs showed that perioperative steroid use could reduce the incidence and severity of dysphagia within 1 year after ACDF, reduce VAS scores within 2 weeks after surgery, and shorten the length of hospital stay without increasing operating time, VAS scores at 6 months and 1 year, and affecting fusion rates.

The principal findings of the present meta-analysis were consistent with those of the previous meta-analysis. Song et al. (36) performed a meta-analysis of six RCTs and two case-control studies and concluded that retropharyngeal steroid use could reduce dysphagia rate, severe dysphagia rate following anterior cervical surgery, without increasing operating time. A meta-analysis of seven RCTs conducted by Garcia et al. (37) concluded that patients treated with corticosteroids intravenously or locally had significantly decreased severity of dysphagia. Yu et al. (38) performed a meta-analysis of 8 RCTs and concluded that perioperative local retropharyngeal steroids could reduce the incidence and severity of dysphagia compared with placebo control. Nevertheless, obvious differences between our meta-analysis and the meta-analysis mentioned above should be taken into account. Most importantly, we dynamically investigated the effect of steroids on dysphagia rate and its severity at 1 day, 2 weeks, 3 months, 6 months, and 1 year after anterior cervical surgery. The above studies may have included too few studies and ignored time as an influencing factor, often taking the last follow-up as the endpoint event. Second, we exhaustively searched various databases with a standardized and detailed search strategy and finally included 14 RCTs of 1,181 patients. The overall risk of bias of the included studies was determined to be low. Third, we performed a subgroup analysis to investigate the effects of intravenous and local steroids. The results showed that there was no significant difference between intravenous and local steroid administration regarding dysphagia rates (*p* = 0.82), fusion rate (*p* = 1.00), and operative time (*p* = 0.10). However, the above studies did not quantitatively compare the efficacy of topical or intravenous administration of the steroids.

From our analysis, the incidence and severity of dysphagia significantly decreased with steroids within 1 year following anterior cervical surgery. Previous reviews have consistently reported the benefit of steroids on dysphagia and its severity. Zadegan et al. (39) reviewed 7 RCTs and 2 non-RCTs, and concluded that the incidence and severity of dysphagia was significantly lower in the steroid group. Cheng et al. (40) reviewed 3 RCTs and 2 retrospective cohort studies, and concluded that local corticosteroid application could reduce the incidence and severity of dysphagia following ACDF. Adenikinju et al. (41) reviewed 5 RCTs and 2 retrospective cohort studies, and concluded that patients received systemic and local steroids benefit from reductions in rate and severity of dysphagia postoperatively. However, our finding is a novelty because we performed a qualitative synthesis of RCTs and discuss dysphagia without the differences in time points. In our subgroup analysis, we only have sufficient data exploring the effect of local and intravenous application of steroids on dysphagia rates at postoperative 1 day and found that there was no significant difference between intravenous and local steroid administration regarding dysphagia rates. This is consistent with the findings from 1 previous systematic review that Garcia et al. (37) performed a high-quality meta-analysis of 7 RCTs and found that there was no significant difference between intravenous and local steroid administration. Further high-quality RCTs are needed to directly compare the effect of local and intravenous application of steroids on dysphagia and its severity.

Many spine surgeons worry that steroids negatively impact bony fusion rates and are reluctant to use steroids. Our results demonstrated that there was no difference in fusion rates at 1-year follow-up between the steroids group and control group, which were consistent with those of prior studies of perioperative steroids (18, 22, 29, 39, 41). Nevertheless, the steroids may hinder early fusion. Jeyamohan et al. (29) reported that fusion rates at 6 months proved to decrease in the steroid group but lost significance at 12 months. In addition, it should be taken into account that the definition of fusion was not the same in these five included studies. Cui et al. (22) considered fusion to be achieved if radiographs demonstrated <1 mm of interspinous motion between flexion and extension or if CT or MRI demonstrated clear evidence of bone bridging from end plate to end plate. Dahapute et al. (23) and Jenkins et al. (28) used a CT scan to confirm fusion without giving a detailed definition of fusion. Jeyamohan et al. (29) considered the spine was fused if bridging osseous trabeculae were observed spanning each operative level without any intervening radiographic lucencies. Similarly, Lee et al. (18) considered that the presence of bony extension into the space between the graft and the absence of segmental motion supported the fusion. Future studies with large sample sizes, uniform standards and longer follow-up time for bony fusion are needed to validate our findings.

Our results showed that a significant decrease regarding VAS score in the steroid group was observed compared with that in the control group in the short-term follow up. Previous studies have demonstrated the benefits of steroid use regarding to direct feelings calculated by the VAS at postoperative 2 weeks (18, 23, 25, 26, 28). In our included RCTs, Dahapute et al. (23) found that VAS score at postoperative 1 day and 2 weeks proved to decrease in the steroid group but lost significance at 2 months. Jenkins found that there existed a significant difference between steroids and control group regarding VAS score at postoperative 1 day and 2 weeks but lost significance at 3 months. Both support the short-term of benefits of steroids on VAS score. Considering the heterogeneity of the results obtained by our quantitative calculation of VAS, it is unsafe to conclude that steroids can reduce VAS score with such a good effect, but it can be inferred that the steroids have a short-term effect in terms of VAS score after surgery. In our subgroup analysis, there existed a significant difference between intravenous and local steroid administration regarding VAS score at postoperative 1 day (Local: WMD = −2.22, 95% CI: −3.03 to −1.42 vs. Intravenous: WMD = −0.10, 95% CI: −0.46 to 0.25). However, in an RCT conducted by Jenkins et al. (28), their results showed that there was no significant difference between intravenous and local steroid administration regarding VAS score. Additionally, when removing the study of Nam et al. (16), the findings for VAS score were consistent with previous analysis. We should interprete the finding with caution and look forward more high-quality RCTs that directly compare the effect of local and intravenous application of steroids VAS score.

In our series, we found that patients receiving steroids had shorter length of hospital stay compared to the control groups. This is consistent with the findings of previous studies (13, 15, 29, 33). This may be explained by the improved symptoms of dysphagia incidence and severity in the steroid group. Next, we investigated the effect of steroids on operation time and the results showed there was no significant difference between groups in operating time, which indicated that steroids do not increase the risk of prolonged surgery. In the included 7 RCTs that reported the detailed operation time, only Kim et al. (30) reported fewer operation time in steroid group compared with control group. It is possible that the operation time in their study was about twice as long as in the other studies, amplifying the effect of steroids on operation time.

Major concerns regarding the use of steroids are steroid-related complications. Despite the reported increased infection rate related with steroid application in general (42, 43), the present meta-analysis showed that there was no significantly increased risk of infections with steroid use in any of the included studies. Esophageal perforation is one of the most dreadful complications of ACDF with an incidence of 0.02%–1.52% (44). Lee et al. (45) cautioned that esophageal perforation was a potential complication of local perioperative steroids in the late post-operative period of ACDF. However, this complication was not reported in any of the included studies. Actually, the two cases reported in the literature of esophageal perforation were both on chronic steroids, therefore, it is uncertain whether the esophageal perforation was directly associated with perioperative steroids. Taken together, steroids application does not increase the risk of early potential complications, but future studies are still necessary to evaluate the potential long-term complication associated with steroids administration.

The current meta-analysis observed some limitations. First, various doses and steroid types were adopted in the included studies, exact dose and type of steroid for desired effect on incidence and severity of dysphagia remains unclear. Though we performed a subgroup analysis by the route of administration (Local vs. Intravenous), it is still insufficient to account for a long-term effect of local and intravenous steroids on dysphagia. Second, even though we included 14 RCTs, only a few were used for quantitative analysis when comparing a specific outcome. This is due to differences in the way dysphagia was assessed and the variety of outcomes reported between studies. Finally, the number of fusion levels also varied across studies, exposing patients to different risks and potentially leading to different responses to interventions. In addition, the Grade results qualities of VAS were low, and dysphagia event, Bazaz stratification of severity of dysphagia, fusion rate, operation time and length of hospital stay were moderate. None of high quality evidence was found in above outcomes. Therefore, further high-quality studies are required to determine which subpopulations are most likely to benefit or not, and more individualized treatment is needed.

## Conclusion

The current meta-analysis demonstrates the benefits of perioperative steroid administration in anterior cervical surgery without increasing the risk of early potential complications. Future high-quality RCTs are warranted to recommend the administration of steroids in anterior cervical surgery.
