# Supplementary material for: Effect of perioperative steroids application on dysphagia, fusion rate, and visual analogue scale (VAS) following anterior cervical spine surgery: A meta-analysis of 14 randomized controlled trials (RCTs)
Source: Front Surg. 2022 Nov 1;9:1040166. doi: 10.3389/fsurg.2022.1040166 (PMC9663824; doi:10.3389/fsurg.2022.1040166)
Supplement: Supplementary file 1 [file Datasheet1.pdf]

Pubmed

#1 ("Glucocorticoids"[Mesh]) OR ((Glucocorticoid[Title/Abstract]) OR (Glucocorticoid Effect[Title/Abstract]) OR (Effect, Glucocorticoid[Title/Abstract]) OR (Glucorticoid Effects[Title/Abstract]) OR (Effects, Glucorticoid[Title/Abstract]))

#2 ("Steroids"[Mesh]) OR ((Steroid[Title/Abstract]) OR (Catatoxic Steroids[Title/Abstract]) OR (Steroids, Catatoxic[Title/Abstract]))

#3 ("Methylprednisolone"[Mesh]) OR ((Metipred[Title/Abstract]) OR (6-Methylprednisolone[Title/Abstract]) OR (6 Methylprednisolone[Title/Abstract]) OR (Urbason[Title/Abstract]) OR (Medro[Title/Abstract]))

#4 ("Dexamethasone"[Mesh]) OR ((Methylfluorprednisolone[Title/Abstract]) OR (Hexadecadrol[Title/Abstract]) OR (Decameth[Title/Abstract]) OR (Decaspray[Title/Abstract]) OR (Dexasone[Title/Abstract]))

#5 ("Adrenal Cortex Hormones"[Mesh]) OR ((Hormones, Adrenal Cortex[Title/Abstract]) OR (Corticosteroids[Title/Abstract]) OR (Corticosteroid[Title/Abstract]) OR (Corticoids[Title/Abstract]) OR (Corticoid[Title/Abstract]) OR (Adrenal Cortex Hormone[Title/Abstract]) OR (Cortex Hormone, Adrenal[Title/Abstract]) OR (Hormone, Adrenal Cortex[Title/Abstract]))

#6 (anterior cervical discectomy and fusion[Title/Abstract]) OR (Anterior cervical surgery[Title/Abstract]) OR (Anterior cervical fusion[Title/Abstract]) OR (ACDF[Title/Abstract]) OR (Anterior Cervical Corpectomy and Fusion[Title/Abstract]) OR (ACCF[Title/Abstract]) OR

#7 #1 OR #2 OR #3 OR #4 OR #5

#8 #7 AND #6

Web of science

#1 TS=(Glucocorticoids OR Glucocorticoid OR Glucocorticoid Effect OR Effect, Glucocorticoid OR Glucorticoid Effects OR Effects, Glucorticoid)

#2 TS=(Steroids OR Steroid OR Catatoxic Steroids OR Steroids, Catatoxic)

#3 TS=(Methylprednisolone OR Metipred OR 6-Methylprednisolone OR 6 Methylprednisolone OR Urbason OR Medro)

#4 TS=(Dexamethasone OR Methylfluorprednisolone OR Hexadecadrol OR Decameth OR Decaspray OR Dexasone OR Dexpak OR Maxidex OR Millicorten OR Oradexon OR Decaject OR Decaject-L.A. OR Decaject L.A. OR Hexadrol)

#5 TS=(Adrenal Cortex Hormones OR Hormones, Adrenal Cortex OR Corticosteroids OR Corticosteroid OR Corticoids OR Corticoid OR Adrenal Cortex Hormone OR Cortex Hormone, Adrenal OR Hormone, Adrenal Cortex)

#6 TS=(anterior cervical discectomy and fusion OR Anterior cervical surgery OR Anterior cervical fusion OR ACDF OR Anterior Cervical Corpectomy and Fusion OR ACCF)

#7 #1 OR #2 OR #3 OR #4 OR #5

#8 #6 AND #7

Embase

#1 Glucocorticoids':ab,ti OR 'Glucocorticoid':ab,ti OR 'Glucocorticoid Effect':ab,ti OR 'Effect, Glucocorticoid':ab,ti OR 'Glucorticoid Effects':ab,ti OR 'Effects, Glucorticoid':ab,ti

#2 Steroid':ab,ti OR 'Steroids':ab,ti OR 'Catatoxic Steroids':ab,ti OR 'Steroids, Catatoxic':ab,ti

#3 Metipred':ab,ti OR '6-Methylprednisolone':ab,ti OR '6 Methylprednisolone':ab,ti OR 'Urbason':ab,ti OR 'Medro':ab,ti
#4 Methylfluorprednisolone':ab,ti OR 'Hexadecadrol':ab,ti OR 'Decameth':ab,ti OR 'Decaspray':ab,ti OR 'Dexasone':ab,ti OR 'Dexpak':ab,ti OR 'Maxidex':ab,ti OR 'Millicorten':ab,ti 'Oradexon':ab,ti OR 'Decaject':ab,ti OR 'Decaject-L.A.':ab,ti OR 'Decaject L.A.':ab,ti OR 'Hexadrol':ab,ti
#5 Adrenal Cortex Hormones':ab,ti OR 'Hormones, Adrenal Cortex':ab,ti OR 'Corticosteroids':ab,ti OR 'Corticosteroid':ab,ti OR 'Corticoids':ab,ti OR 'Corticoid':ab,ti OR 'Adrenal Cortex Hormone':ab,ti OR 'Cortex Hormone, Adrenal':ab,ti OR 'Hormone, Adrenal Cortex':ab,ti
#6 'Anterior cervical surgery':ab,ti OR 'Anterior cervical fusion':ab,ti OR 'ACDF':ab,ti OR 'ACCF':ab,ti
#7 #1 OR #2 OR #3 OR #4 OR #5
#8 #6 AND #7

Cochrane Library

#1 (Glucocorticoid):ab,ti,kw OR (Glucocorticoid Effect):ab,ti,kw OR (Effect, Glucocorticoid):ab,ti,kw OR (Glucorticoid Effects):ab,ti,kw OR (Effects, Glucorticoid):ab,ti,kw
#2 (Steroid):ab,ti,kw OR (Catatoxic Steroids):ab,ti,kw OR (Steroids, Catatoxic):ab,ti,kw
#3 (Metipred):ab,ti,kw OR (6-Methylprednisolone):ab,ti,kw OR (6 Methylprednisolone):ab,ti,kw OR (Urbason):ab,ti,kw OR (Medro):ab,ti,kw
#4 (Methylfluorprednisolone):ab,ti,kw OR (Hexadecadrol):ab,ti,kw OR (Decameth):ab,ti,kw OR (Decaspray):ab,ti,kw OR (Dexasone):ab,ti,kw OR (Dexpak):ab,ti,kw OR (Maxidex):ab,ti,kw OR (Millicorten):ab,ti,kw OR (Oradexon):ab,ti,kw OR (Decaject):ab,ti,kw OR (Decaject-L.A.):ab,ti,kw OR (Decaject L.A.):ab,ti,kw OR (Hexadrol):ab,ti,kw
#5 (Hormones, Adrenal Cortex):ab,ti,kw OR (Corticosteroids):ab,ti,kw OR (Corticosteroid):ab,ti,kw OR (Corticoids):ab,ti,kw OR (Corticoid):ab,ti,kw OR (Adrenal Cortex Hormone):ab,ti,kw OR (Cortex Hormone, Adrenal):ab,ti,kw OR (Hormone, Adrenal Cortex):ab,ti,kw
#6 (anterior cervical discectomy and fusion):ab,ti,kw OR (Anterior cervical surgery):ab,ti,kw OR (Anterior cervical fusion):ab,ti,kw OR (ACDF):ab,ti,kw OR (Anterior Cervical Corpectomy and Fusion):ab,ti,kw OR (ACCF):ab,ti,kw
#7 #1 OR #2 OR #3 OR #4 OR #5
#8 #6 AND #7

ClinicalTrials

Steroids | Studies With Results | Interventional Studies | ACDF

Google Scholar

(Glucocorticoid OR Steroids OR Metipred OR Methylfluorprednisolone OR Hormones, Adrenal Cortex) AND (anterior cervical discectomy and fusion OR Anterior cervical surgery OR Anterior cervical fusion OR ACDF OR Anterior Cervical Corpectomy and Fusion OR ACCF)

Ovid

(Glucocorticoid OR Steroids OR Metipred OR Methylfluorprednisolone OR Hormones, Adrenal Cortex) AND (anterior cervical discectomy and fusion OR Anterior cervical surgery OR Anterior cervical fusion OR ACDF OR Anterior Cervical Corpectomy and Fusion OR ACCF)
